# Supplementary material for: Single-Cell Protein and Ethanol Production of a Newly Isolated Kluyveromyces marxianus Strain through Cheese Whey Valorization
Source: Foods. 2024 Jun 16;13(12):1892. doi: 10.3390/foods13121892 (PMC11203209; doi:10.3390/foods13121892)
Supplement: Supplementary file 1 [file foods-13-01892-s001.zip › foods-2988298-supplementary.pdf]

Supplementary

Table S1. Kinetic data of Kluyveromyces marxianus strain EXF-5288 cultivated in initial lactose concentration of 35.0±2.0 g/L at T= 20.0°C.

Culture configuration	Hours	Biomass, X (g/L)	SCP (g/L)	Lac_{cons} (g/L)	Ethanol (g/L)	Citric acid (g/L)	$Y_{X/Laccons}$ (g/g)	$Y_{SCP/X}$ (g/g)	$Y_{Eth/Laccons}$ (g/g)	$Y_{Cit/Laccons}$ (g/g)
35.0±2.0g/L Shake flasks	24[c]	5.38± 0.43	2.00± 0.03	27.7± 0.8	2.8± 0.1	0.6± 0.2	0.19± 0.01	0.37± 0.02	0.10± 0.00	0.02± 0.00
	97[a]	9.69± 1.58	3.25± 0.40	34.6± 1.6	0.00	5.2± 0.3	0.28± 0.03	0.34± 0.00	0.00	0.15± 0.00
	120[b]	9.45± 1.70	3.66± 0.33	34.6± 1.6	0.00	5.3± 0.3	0.27± 0.03	0.39± 0.03	0.00	0.15± 0.00
	144[d]	8.16± 0.00	3.28± 0.00	34.6± 0.1	0.00	5.6± 0.0	0.24± 0.00	0.40± 0.00	0.00	0.16± 0.00

a=max Biomass, b=max SCP, c= max Ethanol, d= max Citric acid. Culture conditions: growth on aseptic 250 mL flasks at 180 ± 5 rpm, pH= 3.5.

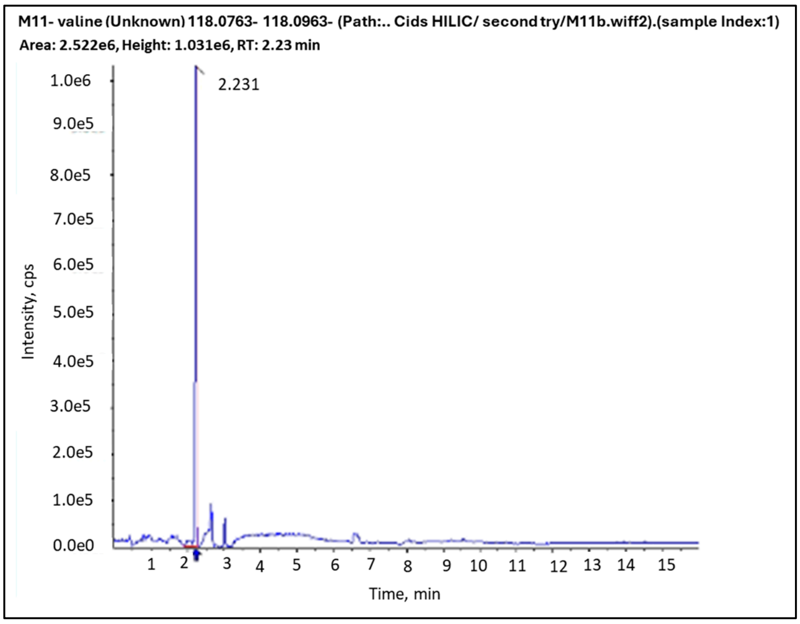

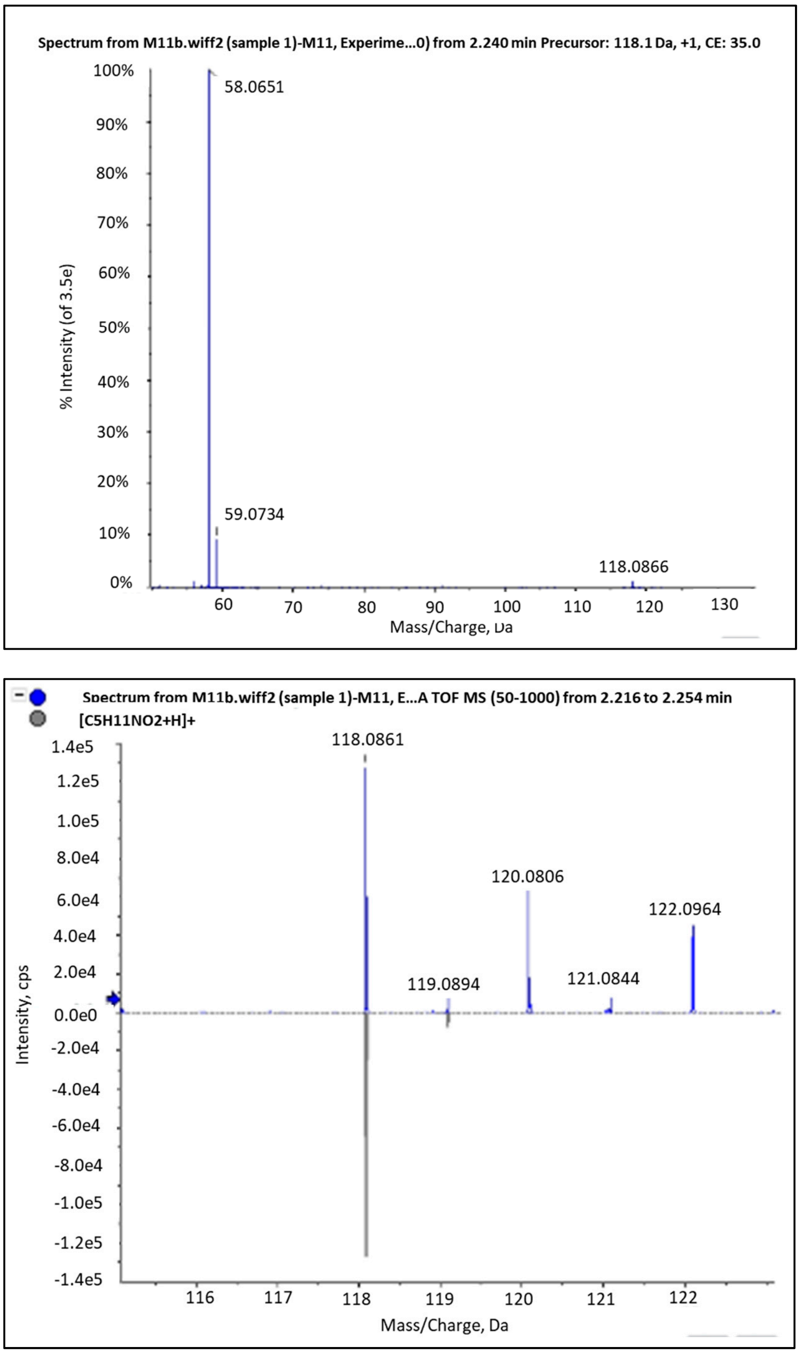
Spectrum from M11b.wiff2 (sample 1)-M11, Experime...0) from 2.240 min Precursor: 118.1 Da, +1, CE: 35.0
% Intensity (of 3.5e)
58.0651
59.0734
118.0866
Mass/Charge, Da
Spectrum from M11b.wiff2 (sample 1)-M11, E...A TOF MS (50-1000) from 2.216 to 2.254 min
[C5H11NO2+H]+
Intensity, cps
118.0861
120.0806
122.0964
119.0894
121.0844
Mass/Charge, Da

(a)

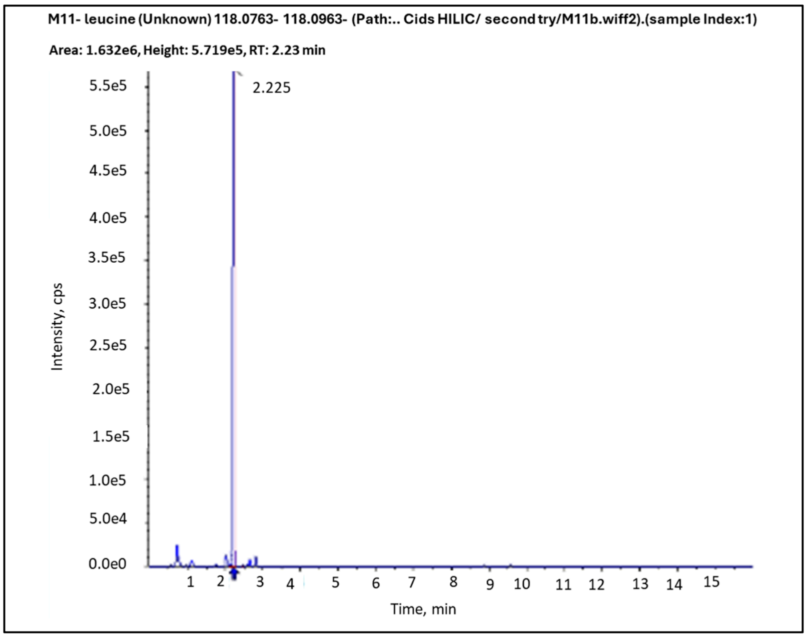

M11- leucine (Unknown) 118.0763- 118.0963- (Path:.. Cids HILIC/ second try/M11b.wiff2).(sample Index:1)
Area: 1.632e6, Height: 5.719e5, RT: 2.23 min
2.225
Intensity, cps
5.5e5
5.0e5
4.5e5
4.0e5
3.5e5
3.0e5
2.5e5
2.0e5
1.5e5
1.0e5
5.0e4
0.0e0
1 2 3 4 5 6 7 8 9 10 11 12 13 14 15
Time, min

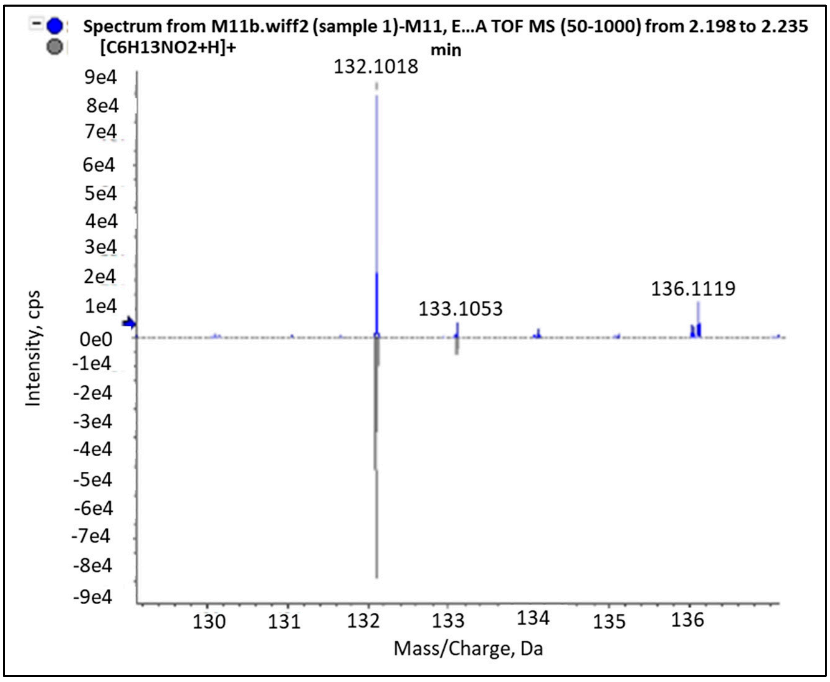

Spectrum from M11b.wiff2 (sample 1)-M11, E...A TOF MS (50-1000) from 2.198 to 2.235 min
[C6H13NO2+H]+
132.1018
133.1053
136.1119
Intensity, cps
9e4
8e4
7e4
6e4
5e4
4e4
3e4
2e4
1e4
0e0
-1e4
-2e4
-3e4
-4e4
-5e4
-6e4
-7e4
-8e4
-9e4
130 131 132 133 134 135 136
Mass/Charge, Da

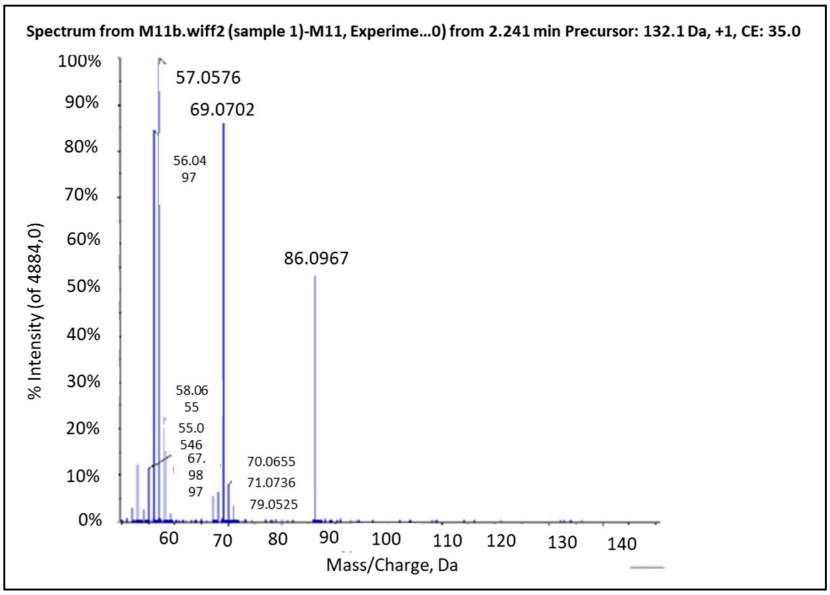
Spectrum from M11b.wiff2 (sample 1)-M11, Experime...0) from 2.241 min Precursor: 132.1 Da, +1, CE: 35.0
57.0576
69.0702
56.04
97
86.0967
58.06
55
55.0
546
67.
98
97
70.0655
71.0736
79.0525
% Intensity (of 4884,0)
100%
90%
80%
70%
60%
50%
40%
30%
20%
10%
0%
60
70
80
90
100
110
120
130
140
Mass/Charge, Da
(b)

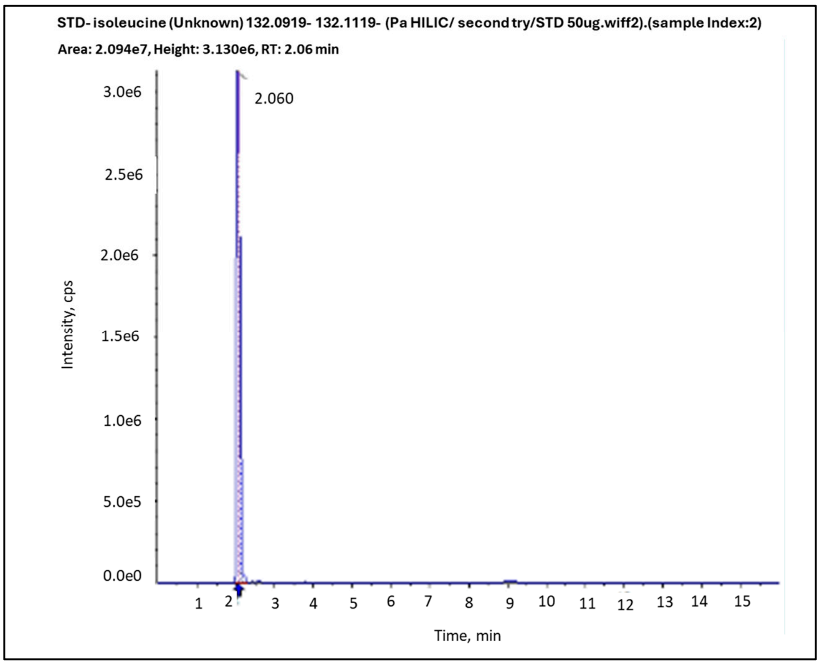
STD- isoleucine (Unknown) 132.0919- 132.1119- (Pa HILIC/ second try/STD 50ug.wiff2).(sample Index:2)
Area: 2.094e7, Height: 3.130e6, RT: 2.06 min
2.060
Intensity, cps
3.0e6
2.5e6
2.0e6
1.5e6
1.0e6
5.0e5
0.0e0
1
2
3
4
5
6
7
8
9
10
11
12
13
14
15
Time, min

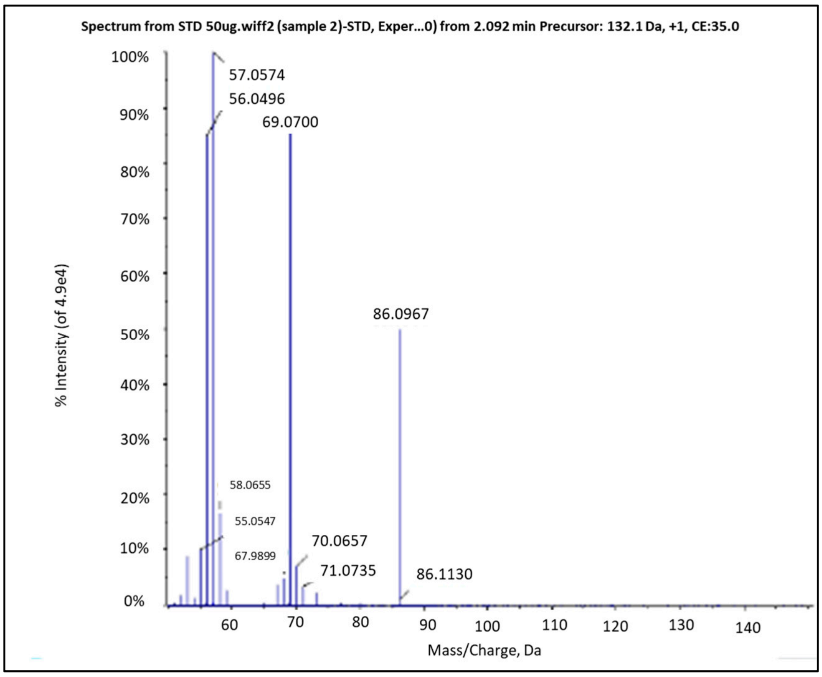
Spectrum from STD 50ug.wiff2 (sample 2)-STD, Exper...0) from 2.092 min Precursor: 132.1 Da, +1, CE:35.0
57.0574
56.0496
69.0700
86.0967
58.0655
55.0547
67.9899
70.0657
71.0735
86.1130
% Intensity (of 4.9e4)
Mass/Charge, Da

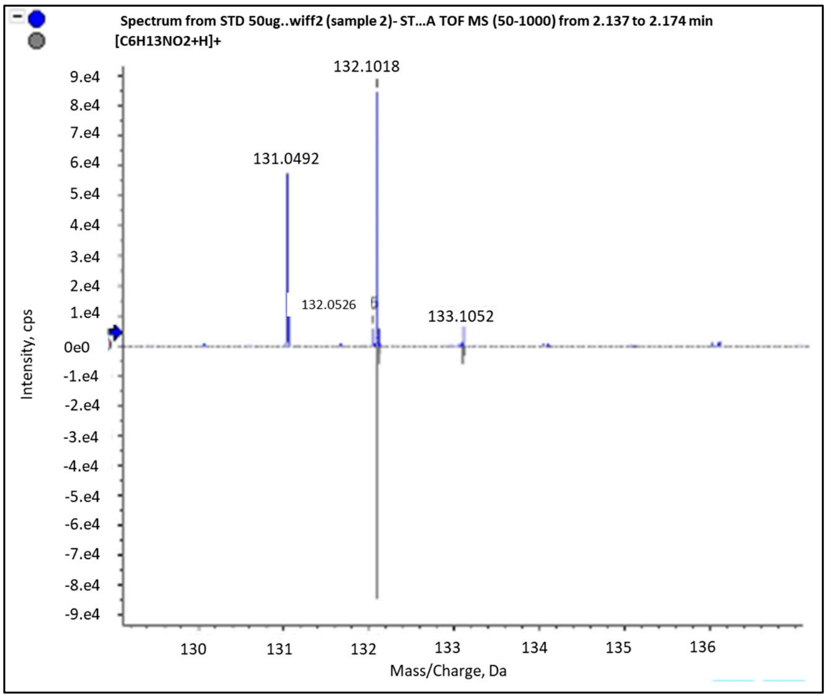
Spectrum from STD 50ug..wiff2 (sample 2)- ST...A TOF MS (50-1000) from 2.137 to 2.174 min
[C6H13NO2+H]+
132.1018
131.0492
132.0526
133.1052
Intensity, cps
Mass/Charge, Da

(c)

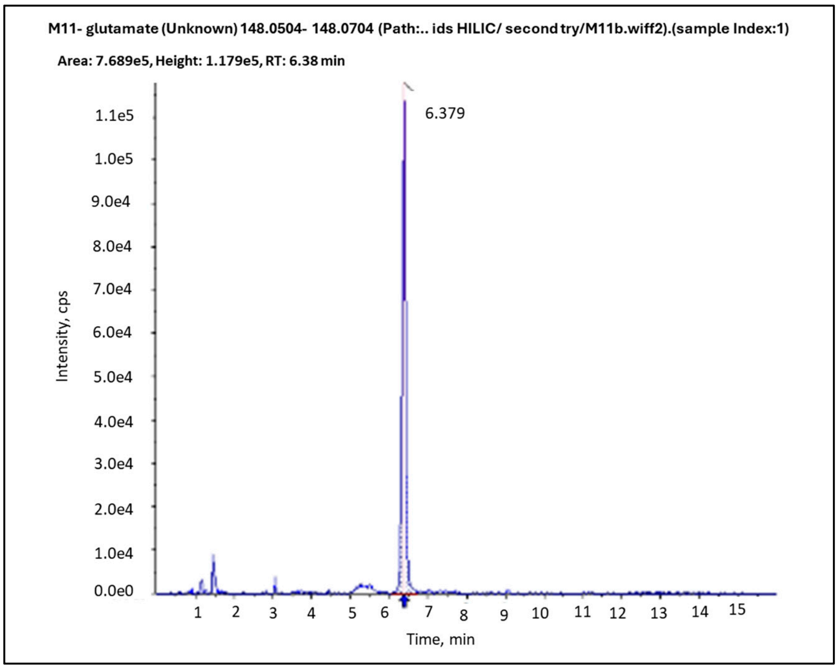
M11- glutamate (Unknown) 148.0504- 148.0704 (Path:.. ids HILIC/ second try/M11b.wiff2).(sample Index:1)
Area: 7.689e5, Height: 1.179e5, RT: 6.38 min
6.379
Intensity, cps
Time, min

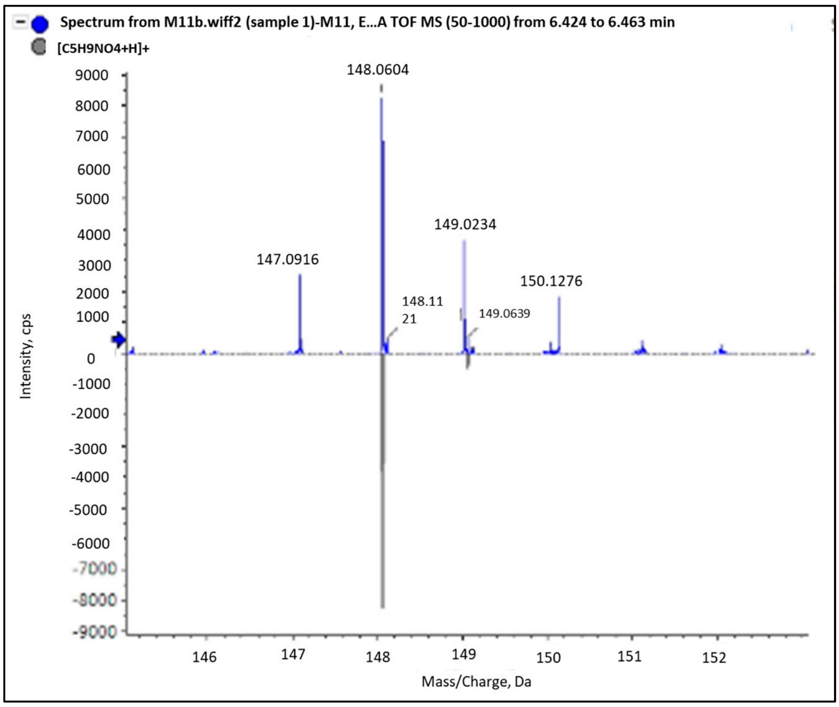
Spectrum from M11b.wiff2 (sample 1)-M11, E...A TOF MS (50-1000) from 6.424 to 6.463 min
[C5H9NO4+H]+
148.0604
149.0234
147.0916
150.1276
148.11
21
149.0639
Intensity, cps
Mass/Charge, Da

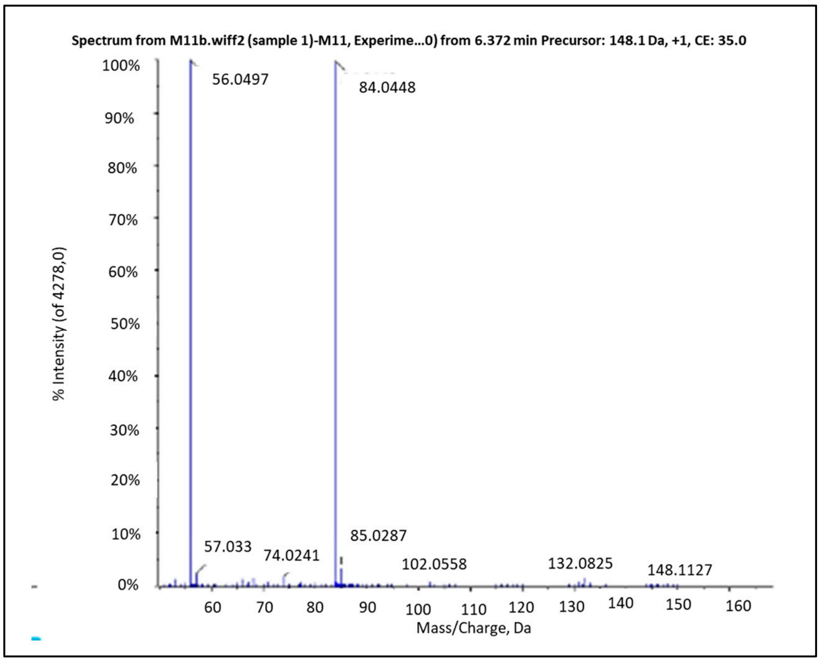
Spectrum from M11b.wiff2 (sample 1)-M11, Experime...0) from 6.372 min Precursor: 148.1 Da, +1, CE: 35.0
% Intensity (of 4278,0)
100%
90%
80%
70%
60%
50%
40%
30%
20%
10%
0%
56.0497
84.0448
57.033
74.0241
85.0287
102.0558
132.0825
148.1127
60
70
80
90
100
110
120
130
140
150
160
Mass/Charge, Da
(d)

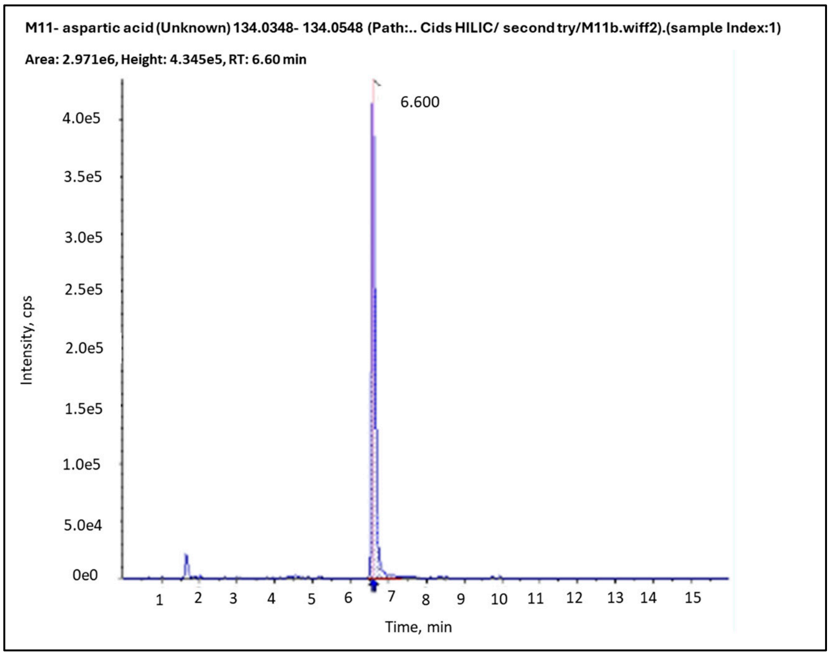
M11- aspartic acid (Unknown) 134.0348- 134.0548 (Path:.. Cids HILIC/ second try/M11b.wiff2).(sample Index:1)
Area: 2.971e6, Height: 4.345e5, RT: 6.60 min
Intensity, cps
4.0e5
3.5e5
3.0e5
2.5e5
2.0e5
1.5e5
1.0e5
5.0e4
0e0
6.600
1
2
3
4
5
6
7
8
9
10
11
12
13
14
15
Time, min

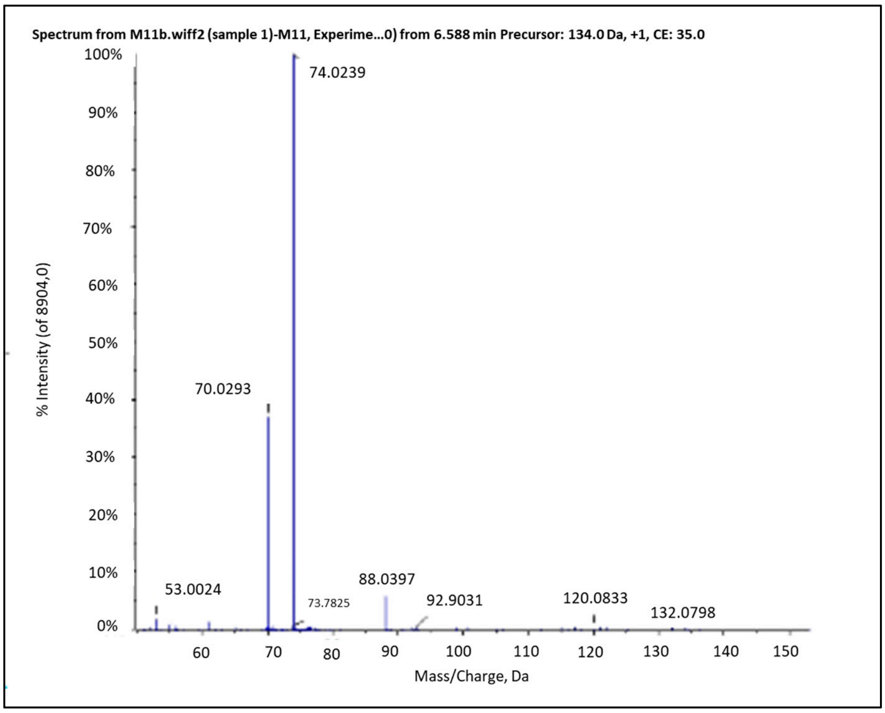

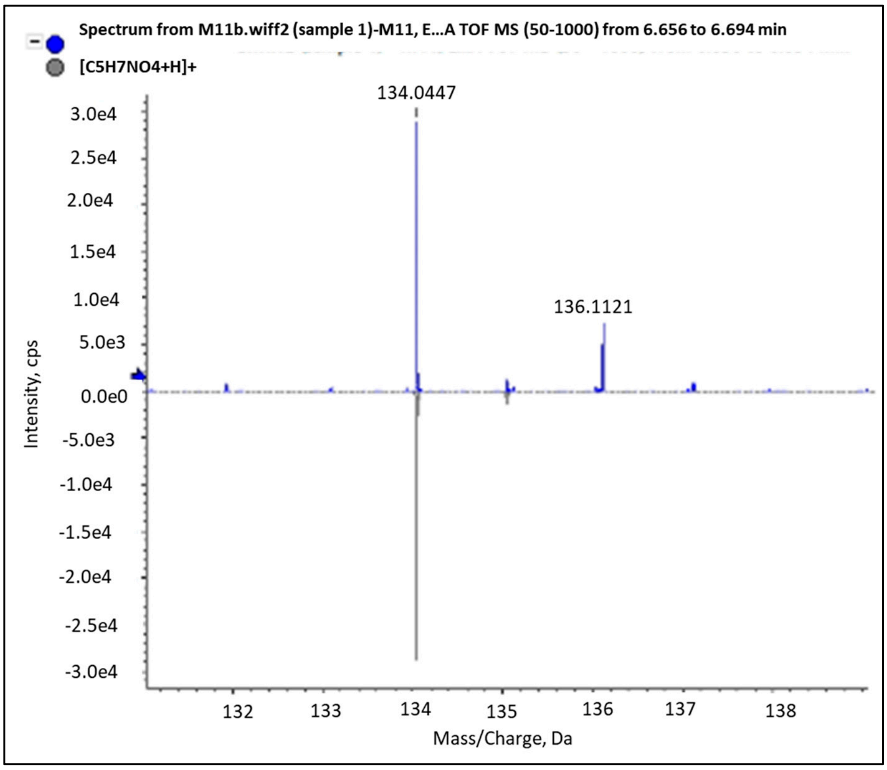

(e)

Figure S1. Extracted Ion Chromatograms, MS and MS/MS spectrum of valine (a), leucine (b), isoleucine (c), glutamic acid (d) and aspartic acid (e).

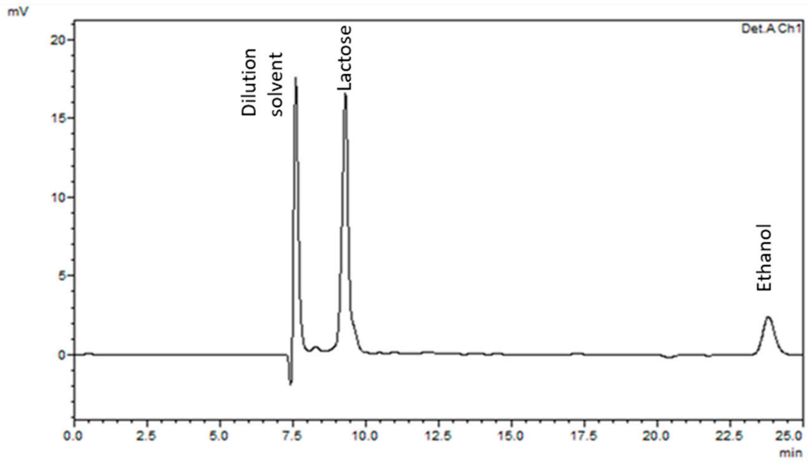
mV
Det.A Ch1
Dilution solvent
Lactose
Ethanol
min

(a)

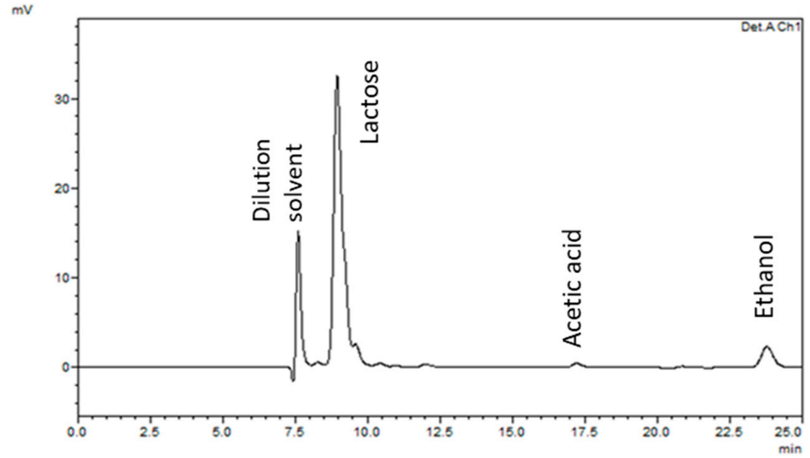
mV
Det.A Ch1
Dilution solvent
Lactose
Acetic acid
Ethanol
min

(b)

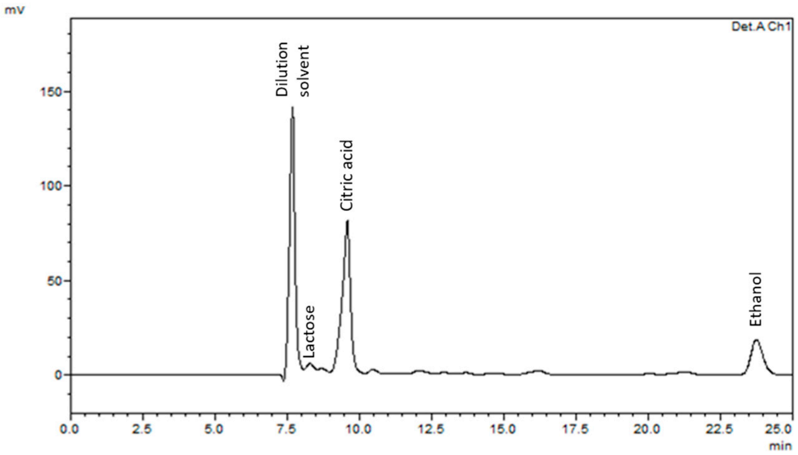

(c)

Figure S2. HPLC chromatograms of experiments conducted at initial lactose of 35.0±2.0 g/L at T= 20.0°C (a), 70.0±5.0 g/L at T=20.0°C (b) and 70.0±5.0 g/L at T=30.0°C (c).
